# Phytotoxic Effects of Catnip (*Nepeta meyeri* Benth.) on Early Growth Stages Development and Infection Potential of Field Dodder (*Cuscuta campestris* Yunck)

**DOI:** 10.3390/plants11192629

**Published:** 2022-10-06

**Authors:** Farid Shekari, Fariborz Shekari, Javad Najafi, Amin Abassi, Zahra Radmanesh, Atle Magnar Bones

**Affiliations:** 1 Department of Plant Production and Genetics, Faculty of Agriculture, University of Zanjan, Zanjan 45371-38791, Iran; 2 Department of Plant Production and Genetics, Faculty of Agriculture, University of Maragheh, Maragheh 83111-55181, Iran; 3 Department of Biology, Norwegian University of Science and Technology (NTNU), 7491 Trondheim, Norway; 4 Department of Plant and Environmental Sciences, University of Copenhagen, Thorvaldsensvej 40, DK-1871 Frederiksberg, Denmark

**Keywords:** allelochemicals, alfalfa infection, alpha-amylase, beta-1,3 glucanase, hydrogen peroxide, seedling growth

## Abstract

Dodder (*Cuscuta campestris* Yunck.) is one of the most devastating parasitic plants, which reduces quantity and quality of crops. The inhibitory effect of catnip (*Nepeta meyeri* Benth.) extracts on germination and some seedling characteristics of the *C. campestris* were investigated in three phases in a laboratory and greenhouse. Aqueous extracts from different organs of *N. meyeri* were used in bioassays. The *N. meyeri* extracts reduced germination percent, root and shoot growth, and dry weight of *C. campestris* seedlings. Moreover, results showed an inhibitory effect of the *N. meyeri* extracts on the activity of alpha-amylase, protease, and beta-1,3-glucanase enzymes in *C. campestris* germinating seeds. Under greenhouse conditions, *C. campestris* seeds were planted with 30-day-old alfalfa plants and irrigated with *N. meyeri* extracts. The application of extracts from different organs of *N. meyeri* reduced emergence percent and length of stem and hampered *C. campestris* attachment to alfalfa. *N. meyeri* extracts also inhibited the activity of antioxidant enzymes and increased the accumulation of hydrogen peroxide and the malondialdehyde in *C. campestris* seedlings. The strongest inhibitory effects were observed from flower, leaf, and stem extracts of *N. meyeri*, respectively. However, after *C. campestris* attachment to alfalfa plants, treatment by *N. meyeri* extracts did not exhibit any effect on infestation efficiency and *C. campestris* growth traits. According to these findings, *N. meyeri* extract, especially from flower and leaf, may be recommended as a potent bio-control agent to control germination and early stage development of *C. campestris*.

## 1. Introduction

Dodder (*Cuscuta campestris* Yunck.) is a flowering parasite plant of the Convolvulaceae family that utilize water and nutrients from the host plant through specialized structures called haustoria [1]. The small seeds of the dodder have a hard cover that, depending on the species, can maintain their vitality in the soil for more than 10 years. Dodder seed germination does not necessarily require the presence of a host plant and can grow between 20 and 30 cm by relying on the maternal seed storages [2]. Dodder control is especially difficult in sugar beet (*Beta vulgaris* L.) and alfalfa (*Medicago sativa* L.) fields in temperate regions [3]. Chemical control is difficult due to the lack of selective herbicides and the dodder’s natural tolerance to common herbicides, such as glyphosate [4]. Moreover, the application of chemical herbicides is associated with environmental contamination [5] and emerging of herbicide-resistant weeds [6]. Therefore, new approaches to weed control using plant-released compounds (allelochemicals) have expanded significantly due to their short half-life, high biodegradability, safety compared to synthetic compounds, and less damage to environment [7]. Allelochemicals enter to natural environment or agricultural systems in various ways, including evaporation of leaf volatiles, leaching of non-volatiles from leaf by rain, secretion from roots, and decomposition of dead plant parts by soil microorganisms [8,9]. The effects of plant compounds on neighbor plant physiology and processes are called allelopathy. In fact, allelopathy is a type of biotic stress that plays an important regulatory role in agroecosystems [10,11]. Essential oils could be major allelochemical compounds that are obtained from plants, such as Lamiaceae family [12]. Due to their allelopathic potential, essential oils can be used as biological agents for weed control [13]. Allelochemical compounds are capable of changing physiological and biochemical processes, including water consumption and use, mineral uptake, leaf development, photosynthesis, amino acid metabolism, protein synthesis, glycolysis, mitochondrial respiration, and ATP synthesis [14].

The genus *Nepeta* belongs to the Lamiaceae family, a subfamily of Nepetoideae and Mentheae tribe [15]. The Lamiaceae family is a source of phenolic compounds with strong antioxidant activity [16] and several studies have reported their allelopathic effects [17,18,19,20]. The genus *Nepeta* includes ~280 species [21], of which 75 species are found in Iran [22]. It has been shown that members of the Lamiaceae family are a rich source of polyphenol compounds and the phytotoxicity of phenolic compounds is due to the disruption and inhibition of certain processes in the germination stage [16,19,23]. Catnip (*N. meyeri* Benth.) is one of the species of this genus. Extraction and characterization of essential oils from the shoot of this plant grown in Anatolia, Turkey, resulted in identification of fifteen compounds, of which 99% were nepetalactone with a high content of oxygen monoterpenes [19]. Mutlu and coworkers [19] hypothesized that *N. meyeri* could have significant allelopathic potential because its presence in natural habitats does not allow other wild plant species to grow and germinate. The inhibition of seed germination and seedling growth due to the application of *N. cataria* extract on various plant species (*Hordeum spontaneum* L., *Taraxacum officinale* L., *Avena fatua* L., *Lepidium sativum* L., *Nepeta cataria* L., *Ocimum basilicum* L.) has been reported [23]. These effects, together with the herbicidal properties of *N. meyeri* on the germination of the tested plants, have proved to be due to the high content of oxygenated monoterpenes, especially 4aα, 7α, 7αβ-nepetalactone [24].

Allelochemicals generated biotic stress can affect target plants [10]. Like other stressors, plants respond to the stress of allelochemicals in a variety of ways. Numerous studies have reported increased activities of antioxidant enzymes and lipid peroxidation of membranes upon application of allelochemical compounds [17,25,26]. During allelopathic stress, production of reactive oxygen species (ROS), such as superoxide, hydrogen peroxide and hydroxyl radicals, significantly increases [26,27,28]. The elevation of ROS causes damage to cell membranes, DNA, proteins, and lipid peroxidation, which can eventually trigger the programmed cell death [27]. In turn, plant cells have evolved a sophisticated defense mechanism to deal with such situations that mainly rely on antioxidants and antioxidant enzymes [29]. 

In this study, we aimed to evaluate the potential allelopathic effects of aqueous extracts from the various organs of *N. meyeri* on germination, establishment, and some biochemical properties of *C. campestris* germinating seeds and seedlings in vitro and in vivo. In addition, the effectiveness of the *N. meyeri* extracts in control of infestation of alfalfa, as a very sensitive host plant, was evaluated before and after parasitation by *C. campestris.*

## 2. Results

### 2.1. Effects of N. meyeri Extracts on C. campestris Seed Germination and Seedling Establishment

Potential effect of *N. meyeri* extracts on the germination of *C. campestris* seeds was tested. Extracts from different parts exhibited differential inhibitory effects on the germination of *C. campestris* seeds. The highest inhibitory effects on germination were observed for *C. campestris* seeds treated with flower extract followed by treatment from leaf extract. The lowest effect on germination rate was recorded for the seeds treated with root extract, which did not differ significantly from the control group treated by distilled water (Table 1).

In addition to germination, the length of the roots and stems of *C. campestris* seedlings were found to be affected by application of extracts from different organs of *N. meyeri*. Similarly, the lowest values were recorded after flower extract treatment, while the highest root and shoot length were observed in the control (Table 1). After flower extract treatment, leaf extract showed the highest inhibitory effect on the growth of various parts of the *C. campestris*. Since the root of the *C. campestris* is very short, variations in this trait after different treatments were low. Similar to the previous traits, the dry weight of the *C. campestris* seedlings was significantly reduced by the application of *N. meyeri* extract (Table 1). The lowest dry weight of seedlings was observed after flower and leaf extract treatments (Table 1). 

### 2.2. Applications of N. meyeri Extracts Affect Activities of Major Enzymes Involved in the Regulation of Seed Germination

**Figure 1:**
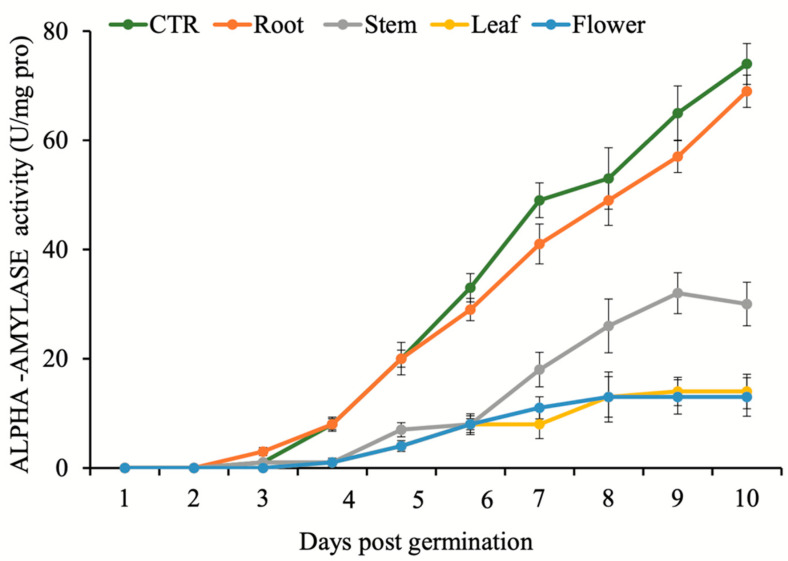
Effect of *N. meyeri* extracts on alpha-amylase activity in germinating *C. campestris* (dodder) seeds. Dodder seeds were treated either by distilled water (CTR) or extracts from different *N. meyeri* plant parts. Error bars represent standard errors calculated from three biological replicates.

**Figure 2:**
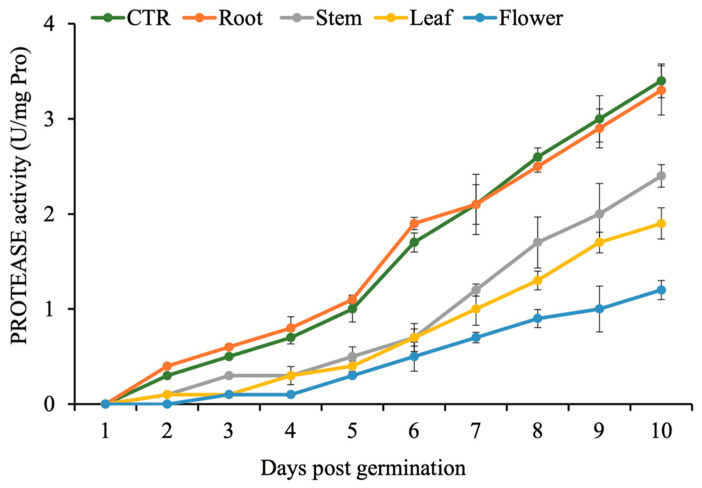
Effect of *N. meyeri* extracts on protease activity in germinating *C. campestris* (dodder) seeds. Dodder seeds were treated either by distilled water (CTR) or extracts from different *N. meyeri* plant parts. Error bars represent standard errors calculated from three biological replicates.

**Figure 3:**
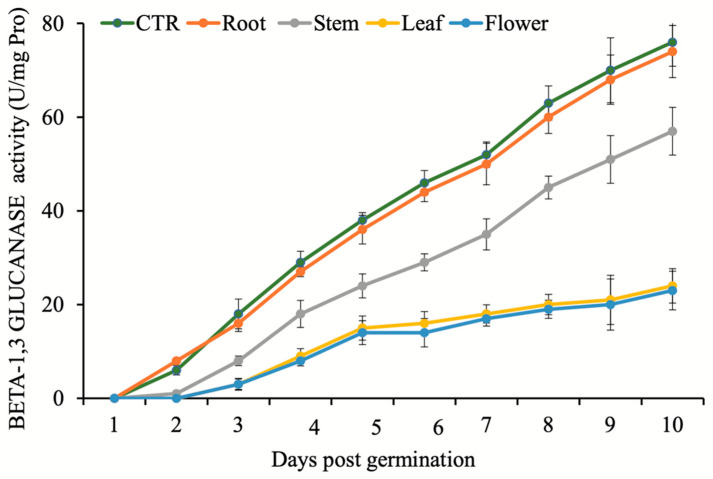
Effect of *N. meyeri* extracts on beta-1,3 glucanase activity in germinating *C. campestris* (dodder) seeds. Dodder seeds were treated either by distilled water (CTR) or extracts from different *N. meyeri* plant parts. Error bars represent standard errors calculated from three biological replicates.

The kinetics of alpha-amylase, protease, and beta-1,3-gluconase enzymes activities in *C. campestris* seeds were monitored over ten days upon treatments by *N. meyeri* extracts (Figure 1, Figure 2 and Figure 3). The highest inhibitory effect on alpha-amylase activity was recorded in the seedlings treated by flower extract, while the lowest repression were recorded in seedlings treated by the root extract (Figure 1). During first three days, no activity was detected upon treatments. However, at day four and following days, a sharp increase in alpha-amylase activity was observed for the seeds treated by root extract and control, while the other treatments exhibited strong inhibitory effect on the activity of this enzyme. Alpha-amylase activity for *C. campestris* seeds treated by leaf and flower extracts was remained steady after day seven (Figure 1).

Similarly, no activity was recorded for protease during first four days after treatments. The lowest protease activity was observed in seedlings treated by flower extracts, while the highest was observed after root extract treatment and control (Figure 2). Kinetics of beta-1,3-glucanase activity was very similar to the protease activity profile. Lowest activities were observed in the seedlings treated by flower and leaf extracts, while the highest were recorded for the seeds treated by root extract and control (Figure 3). A comparison between flower, leaf, stem, and root extracts showed that *N. meyeri* root and stem extracts had less potential to prevent germination and growth of *C. campestris*. Moreover, it was indicated that in germination stage, the highest decrease in *C. campestris* performance belonged to the flower extract treatment, and then to the leaf extract treatment (Table 1).

### 2.3. Effects of N. meyeri Extracts on Parasitation of Alfalfa Plants by C. campestris during Early Phase of Attachment

Similar to the germination stage, the emergence of *C. campestris* seedlings was reduced by application of *N. meyeri* extracts with one exception. There was no significant difference between root extract and control treatment, however, a significant difference was observed between flower and leaf extracts (Table 2). The comparison of extracts from different organs of *N. meyeri* plant showed that flower extract had the highest repressing effect on the emergence of *C. campestris* seedlings. Furthermore, the application of flower extracts increased the number of abnormal seedlings. Flower extract-treated *C. campestris* had a short and thick stem, and a curvature at the tip of the stem, which hinders *C. campestris* attachment to the host plant. None of the emerged *C. campestris* seedlings treated by flower extract were able to attach to the alfalfa plants. The treatment of *C. campestris* with *N. meyeri* leaf or stem extracts, resulted in a fraction of seedlings connected to the alfalfa host plants. The attachments were very weak and *C. campestris* and alfalfa stems could easily be separated. Compared to the control, stem length and stem dry weight of *C. campestris* after treatment by *N. meyeri* extracts were reduced (Table 2). The root extract had no significant effect on the emergence percent of the *C. campestris* but reduced the stem length and dry weight. 

### 2.4. Effects of N. meyeri Extract on ROS Production and Scavenging Enzymes

**Figure 4:**
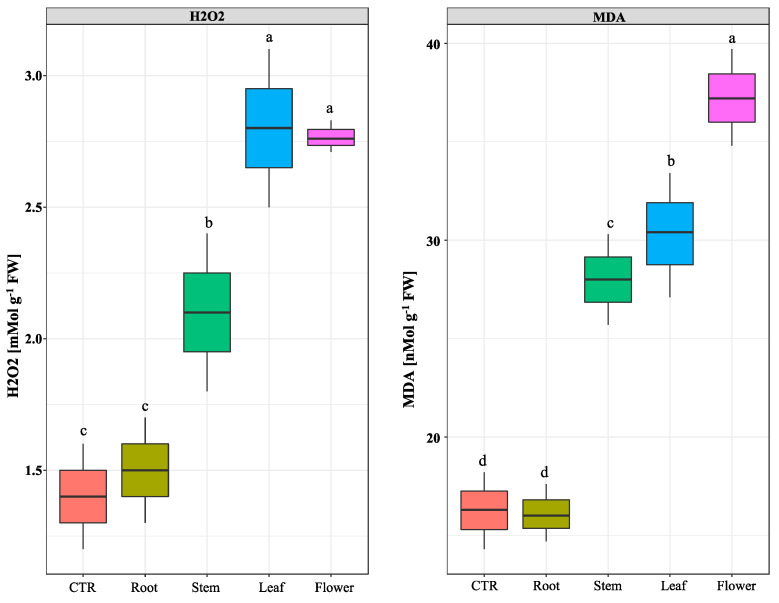
Effect of *N. meyeri* extracts on oxidative stress in *C. campestris* (Dodder) plants. Dodder plants were treated either by distilled water (CTR) or extracts from different parts of *N. meyeri*. Production of H_2_O_2_ (**left**) and MDA (**right**) were quantified. Different letters indicate statistically significant differences calculated by Duncan multiple mean comparison method (n = 3, *p*-value < 0.05).

It has been shown that allelochemicals can induce oxidative stress in treated plants [14,30]. To counteract, targeted plants deploy a wide range of antioxidants and free radicle scavenging enzymes to eliminate or neutralize free radicals. The observed effects of *N. meyeri* extracts on *C. campestris* plants growth and infestation potentials promoted us to measure the production of hydrogen peroxide (H_2_O_2_) and activity of the most known free radicle scavenging enzymes in *C. campestris* plants. As a first assay, we investigated the production of (H_2_O_2_) and malondialdehyde (MDA) upon treatments. The highest level of (H_2_O_2_) accumulation in *C. campestris* plant was observed after flower and leaf extract treatments, while the lowest was related to the plants treated by root extract and control treatments (Figure 4). Similarly, the quantification of malondialdehyde concentration of *C. campestris* seedlings revealed the highest effect for flower extract treatment, while the root extract treatment showed no effect (Figure 4).

**Figure 5:**
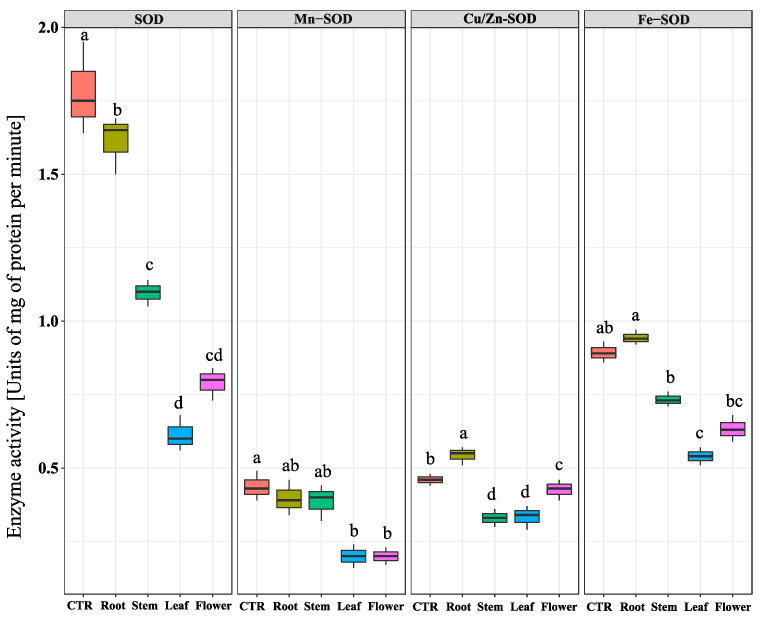
Effect of *N. meyeri* extracts on SOD production in *C. campestris* (dodder) plants. Dodder plants were treated either by distilled water (CTR) or extracts from different part of *N. meyeri.* Different letters indicate statistically significant differences calculated by Duncan multiple mean comparison method (n = 3, *p*-value < 0.05).

**Figure 6:**
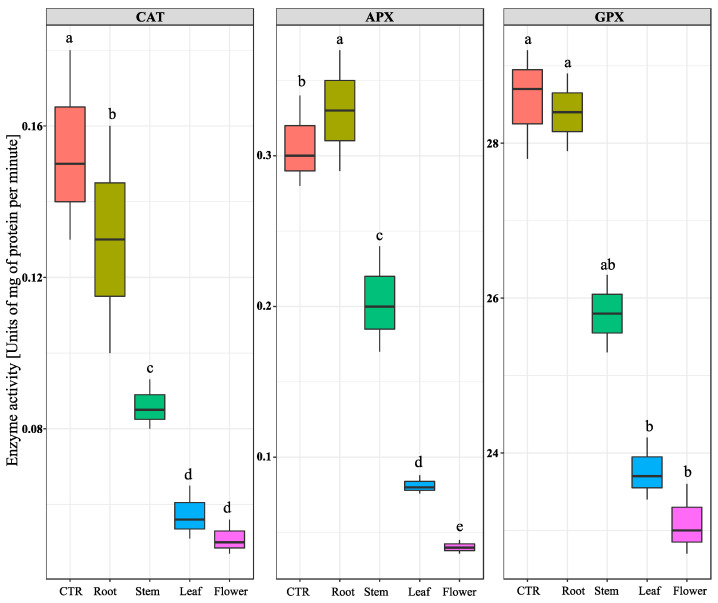
Effect of *N. meyeri* extracts on antioxidant enzymes in *C. campestris* (Dodder) plants. Dodder plants were treated either by distilled water (CTR) or extracts from different parts of *N. meyeri.* Different letters indicate statistically significant differences calculated by Duncan multiple mean comparison method (n = 3, *p*-value < 0.05).

To draw a picture about the oxidative state of the *C. campestris* plants treated with *N. meyeri* extracts, the total superoxide dismutase (SOD) activity and its variants were measured. Results showed that the total SOD activity in plants treated by leaf and flower extracts was dramatically reduced compared to the control. The highest inhibitory effect on Mn-SOD isozyme activity recorded after leaf and flower extracts treatments. A similar trend was observed for the activity of Fe-SOD after treatment by leaf and flower extracts. Regarding to the activity of Cu/Zn-SOD isozyme, the highest repression effects were observed in the *C. campestris* plants treated with stem, leaf, and flower extracts (Figure 5). In addition to SODs, activities of other major ROS scavenging enzymes, including catalse (CAT), ascorbate peroxidase (APX), and glutathione-peroxidase (GPX) were also measured. The quantification of catalse activity showed that treatment by flower and leaf extracts caused about 60% reduction, compared to the control, while root extract treatment had no effect. The lowest activity of APX enzyme in *C. campestris* plants was observed after flower extract treatment, while the highest amount was related to the root extract treatment. Similarly, the measurement of GPX activity in *C. campestris* plants after different treatments, showed significant inhibitory effects after treatment by stem, leaf, and flower extracts (Figure 6).

### 2.5. Effects of N. meyeri Extracts after Attachment of C. campestris to Alfalfa Plants 

In order to evaluate the effects of different treatments on *C. campestris* plants after attachment to alfalfa, we sprayed the plants with *N. meyeri* extracts after establishment. Surprisingly, the spraying of the *N. meyeri* extracts exhibited neither any effect on the degree of attachment nor any visible damage on *C. campestris* after attachment. After all treatments, alfalfa plants showed similar status in terms of infestation degrees and severity of attachment. The dry weight of the *C. campestris* shoots, alfalfa stems and leaves were not affected by spraying with *N. meyeri* extracts. These findings point out that application of *N. meyeri* extracts have no significant biological effect on *C. campestris* after attachment to the host plants (Table 3).

## 3. Discussion

Allelochemicals alter plant’s normal growth and development by a wide range of actions on physiological processes. Plants crude extracts are composed of hundreds of different complex chemicals that have several phytotoxic effects on neighboring canopy. Since identification of each individual compound is challenging, the whole-plant bioassays and physiological tests of compounds in crude extract is a feasible approach to investigate allelochemicals mode of actions and their target pathways. Furthermore, allelopathy is a result of synergistic and antagonistic interactions among several compounds in the plant extracts [30,31]. Among different plants species as potential biocontrol agents of dodder, steam distillation and hot water extracts of catnip (*Nepeta meyeri* Benth.) have been studied. In this study, we used aqueous extracts from different organs of *N. meyeri* as an agent to control seed germination and seedling establishment of *C. campestris*. Previous studies showed that even cold water extracts from this plant have very strong effects on treated chicken’s behaviors [32]. *N. meyeri* extracts showed inhibitory effects on germination and growth of *C. campestris* seedlings and reduced or inhibited the remobilization of substances from the seeds of *C. campestris* to the stem and root. Compared to the control, the treatment of *C. campestris* seeds with *N. meyeri* extract significantly reduced dry weight in *C. campestris* seedlings (Table 1). Inhibitory effects of allelochemicals extracted from *N. meyeri* on germination, growth, and dry weight of seedlings of some weed species [19,24] and crops [23,28] have been investigated. These compounds inhibit seed germination by inducing oxidative stress, as well as restricting the remobilization of seed storage to embryo axis through inhibiting of alpha-amylase enzyme activity and gibberellic acid (GA) synthesis [33,34,35]. The observed effect of essential oils can also be due to the increased levels of lipid peroxidation and hydrogen peroxide concentration [19].

Alpha-amylase is responsible for the breakdown of starch in germinating seeds [36]. According to Xie et al. [37] and Ogawa et al. [38], the production of alpha-amylase in the aleurone layer is strictly controlled by embryonic gibberellins. Detailed molecular analysis revealed that the rate of gibberellin biosynthesis decreases sharply after *N. meyeri* extract application [19]. Consistent with this report, our data showed that the activity of alpha-amylase in *C. campestris* seeds was strongly inhibited after application of the *N. meyeri* extracts from leaf and flower (Figure 1). We measured the total proteases activities during germination to investigate the biological effects of *N. meyeri* extracts on this critical stage. The highest inhibitory effect of *N. meyeri* extracts was observed in the *C. campestris* seeds treated by flower extracts (Figure 2). Beta-1,3-glucanase is another important enzyme in the regulation of germination. Before germination, this enzyme accumulates in the micropillar of the seed endosperm [39,40] and plays an important role in the hydrolysis of cell wall compounds and in the process of endosperm loosening at the radicle exit site [41]. The application of extracts from different organs of *N. meyeri* showed that the activity of beta-1,3-glucanase enzyme in *C. campestris* seeds was significantly affected (Figure 3). It has been shown that the completion of the germination process depends largely on beta-1,3-glucanase activity [42]. 

In both trials of germination assays in the laboratory and greenhouse, the length of stem and root of *C. campestris* were reduced (Table 1 and Table 2). The reduction in stem length can be considered simply from a physical point of view, as a reduction in the possibility of collision and attachment of *C. campestris* with the host plant. It can also be explained by the reduced capacity of the hypha production. Comparably, the reduced length of the root can affect the establishment of *C. campestris* plant in the early stages and restricts water uptake. However, the application of *N. meyeri* extracts did not exhibit any inhibitory effects after dodder attachment to the host alfalfa plants (Table 3). This observation points towards the possibility that early stage application of *N. meyeri* extracts can hinder the physical attachment of dodder to the host plant, probably through alteration of oxidative stress response in seedlings. The observed germination, establishment, and growth phenotypes can be explained by the recorded kinetics of alpha-amylase, protease, and beta-1,3-glucanase enzymes (Figure 1, Figure 2 and Figure 3). Studies have shown that some allelochemical compounds can indirectly cause root cell death and alter hormonal balance during the germination phase by facilitating the production of ROS, which acts as signaling molecules [43]. On the other hand, the essential oils of medicinal and aromatic plants are mainly composed of terpenes, terpenoids, phenylpropanoids, and aliphatic compounds. Phenolic compounds are able to interfere with the normal growth and development of all parts of the plant by preventing root cell division, root elongation, and root ultrastructural alterations [44,45,46]. It has been shown that allelopathic effect of *N. meyeri* extract on the root growth of some plants correlated to the applied concentration [27]. 

Plants have a highly efficient defense system that can eliminate or neutralize free radicals to counteract the oxidative stress [46]. This defense system includes antioxidant enzymes, such as superoxide dismutase (SOD), catalase (CAT), glutathione peroxidase (GPX), and ascorbate peroxidase (APX). The measurement of antioxidant enzymes activities in stems of *C. campestris* after *N. meyeri* extract treatments indicated an extensive variation in the activity of three enzymes and revealed a significant effect of allelopathic stress. By reducing the activity of total antioxidant enzymes, such as SOD and its isozymes, APX, CAT, and GPX under *N. meyeri* extracts (Figure 5 and Figure 6), it seems that delicate balance between the production of reactive oxygen species and scavenging mechanisms are impaired. In line with our observations, Mutlu et al. [19] reported that essential oils extracted from *Nepeta meyeri* increased the activity of catalase in all studied weed species but reduced the activity of SODs. Previous studies have reported oxidative stress due to the effects of allelochemicals on tomato seedlings [47]. The spraying of peppermint allelochemical compounds at low concentrations on tomato seedlings showed promoting effects in growth traits, while higher concentrations of these compounds induced stress in the plant and increased generation of ROS [10]. Hence, in the treated plants, the activity of antioxidant scavenging enzymes and proline content were elevated to attenuate the damages caused by the accumulated ROS compounds.

The rates of lipid peroxidation were used as a marker to assess the state of the cell damage under stress conditions. We used this criterion to evaluate the degrees of cell damages in *C. campestris* plants after treatments by extracts from different organs of *N. meyeri*. The analysis of malondialdehyde (MDA) showed that treatment by extracts from aerial organs of *N. meyeri* significantly increased MDA levels (Figure 4). Damage to cell membranes can be the result of elevated levels of hydrogen peroxide (H_2_O_2_) in response to treatments using *N. meyeri* extracts and a decrease in the activity of important antioxidant enzymes (Figure 4 and Figure 5). Studies have reported increased activities of antioxidant enzymes and lipid peroxidation of membranes as a result of allelochemical compounds applications [17,25,26]. The toxic levels of ROS can affect membrane permeability, causing DNA and protein damage and lipid peroxidation and ultimately trigger programmed cell death [46]. Many allelochemicals have toxic consequences to plant cells that must be detoxified. This process and the response of plant cells to oxidative agents results to increased activity of antioxidant enzymes [47]. Observed increase in the production of H_2_O_2_, the most important O_2_^−^ superoxide radical, indicated that *N. meyeri* extract has caused an overproduction of superoxide radicals and oxidative stress in *C. campestris* plant (Figure 4). Mutlu et al. [19] reported that essential oils extracted from *Nepeta meyeri* increased lipid peroxidation rates and H_2_O_2_ concentrations in treated weed species. The accumulation of hydrogen peroxide in weeds increases lipid peroxidation and oxidative stress, resulting in metabolic disorders. 

In both laboratory and greenhouse experiments, the most allelopathic effects were observed in flower and leaf extracts. Stem extract was less effective than flower and leaf extracts, while no significant effects were observed for root extract. Oueslati [48] reported that leaf extract of durum wheat cultivars was more effective than the root extract. The difference between the extracts of different parts of a plant is due to differences in the concentration of active compounds or the existence or absence of some compounds. According to Basalingamma et al. [49], inhibitory or stimulant effects of the extracts may be related to the quantity of allelochemical compounds and their composition. It is obvious that the composition of allelochemicals are significantly varied between species, environmental conditions, genetic background, harvest time, extraction method, and developmental stages [50,51,52]. Further analytical studies on the composition of extracts using different methods from *N. meyeri* flowers, leaves, stems, and roots of *N. meyeri* are recommended to determine the most effective combination to reduce the growth and parasitation potential of *C. campestris*.

## 4. Materials and Methods

### 4.1. Plant Materials 

Inflorescences of *C. campestris* with fresh seeds were collected from alfalfa field of Zanjan University (Zanjan, Iran), located in 36°, 41′ north and 48° and 27′ east in late summer 2016. Due to high water content, seeds were dried at room temperature for 15 days before storage. Seeds of dodder have dormancy. The highest level of dormancy is reported for seeds separated from the mother plant [53]. In order to break dormancy, seeds were stored in closed plastic bags at 4 °C for one year. In order to evaluate effects of catnip (*Nepeta meyeri*) extracts from different organs and tissues on the *C. campestris* germination and post germination development, we have collected fully developed *N. meyeri* plants from their natural habitat for allelochemical extraction and bioassays. In the next fall, *N. meyeri* plants, which began blooming in September, were collected from the Mishoo rangeland area (East Azarbaijan, Iran), located in 38° and 25′ north and in 45° and 46′ east. Plants were collected carefully in plastic bags and transferred to the laboratory. In the laboratory, plants were washed with tap water. Root, stem, leaf, and flower were separated and cut into 1 cm pieces. The parts were mixed with double-distilled water in a ratio of 1:5 (*w*/*w*). Tissues were crushed and refined by passing them through three layers of Whatman papers to get homogenous suspensions. Finally, the solutions were pasteurized in a water-bath at 70 °C for 15 min. Extracts were stored at 4 °C for subsequent applications.

### 4.2. In Vitro and In Vivo Bioassays

The present study was conducted in three separate phases:

Phase 1: Petri dish bioassays: dodder seeds were disinfected with 5% sodium hypochlorite solution for 5 min and washed with sterile distilled water. Fifty seeds were placed in 10 cm diameter Petri dishes. The experiment was performed in a completely randomized design in three replicates. Treatments included root, stem, leaf, and flower extracts, along with distilled water as control. Ten ml of each extract was added to Petri dishes at the beginning of experiment. Three ml of extracts were added to Petri dishes in the intervals of three days. Petri dishes were incubated for 10 days at 20 ± 2 °C. At the end of the day ten, the number of germinated seeds was counted, and length of roots and stems were measured. After removing cotyledons, seedlings were dried at 70 °C for 24 h in the oven and dry weight of seedling were determined. Seeds were considered germinated when the radicle and plumule elongated at least to two mm. The germination percent was calculated using the following equation:

GP = (G_N_/S_N_) × 100 (1)

where G_N_ is the total number of germinated seeds on the last day of experiment and S_N_ is the total number of sown seeds.

Enzymes assay involved in regulation of seed germination: In parallel, one day after placing seeds in the Petri dish, the activity of alpha-amylase (EC 3.2. 1.1.), protease (EC 3.4.21.112) and beta-1,3 glucanase (EC 3.2.1.39) enzymes were measured according to Roberts and Whitehouse [54], Kunitz [55], and Abeles and Forrence [56] methods, respectively.

Phase 2: Whole plant bioassay and irrigation treatment: Disinfected alfalfa seeds ‘Ghareh Yonjeh’ cultivar were planted in eight-liter pots filled with a mixture of field soil, manure and sand (6:3:1 ratio) at a density of ten plants per pot. Thirty days after establishment of alfalfa plants, 50 seeds of disinfected *C. campestris* were sown at a depth of approximately one centimeter. The pots were kept in the greenhouse under 16 h/8 h light–dark regime with day temperature of 24–28 °C and night temperature of 12–16 °C in three replicates in a completely randomized design. After sowing of *C. campestris* seeds, pots were irrigated with the same extracts and treated, as mentioned in the previous section, with an interval of 3 to 4 days. Irrigation was done for each pot until they reached to the field capacity. Extracts were used to irrigate the pots until end of the experiment. The experiment continued for the next 45 days. The emerged seedlings percent was calculated by substituting the number of emerged seedlings instead of germinated seeds (Equation (1)) [57]. At the end of the experiment, the number of *C. campestris* plants that were able to attach their hypha into the alfalfa plants and fully connect with *C. campestris* plants was considered as the infection percent (Equation (2)).

IP = (IS/ES) × 100(2)

where IP is the infection percent, IS represents the number of *C. campestris* seedlings attached to alfalfa plants, and ES is the number of emerged *C. campestris* seedlings.

At the end of the experiment, *C. campestris* plant materials were collected and divided in two parts. One part from each treatment of the collected *C. campestris* plants from the pots were dried by incubating at 75 °C for 48 h to measure the dry weights. The second part was used for enzymatic assays, as described in the following section. The data were obtained from three independent replications (ten plants pooled for each replication).

Phase 3: Whole plant bioassay—spraying treatment: Alfalfa seeds were planted in eight-liter pots and kept inside the greenhouse under 16 h/8 h light–dark regime with day temperature of 24–28 °C and night temperature of 12–17 °C. After emergence and establishment of alfalfa seedlings, *C. campestris* seeds were sown in each pot and, as described in previous stage, ten alfalfa plants were kept in each pot and irrigated with tap water. After *C. campestris* stems attachment to the alfalfa plants, the *N. meyeri* extracts were used to spray alfalfa and *C. campestris* plants three times per day for thirty days. Spraying the plant extract was done with a hand spray. Thirty days after observation of the first signs of infection and attachment, alfalfa and *C. campestris* plants were removed from the pots, *C. campestris* stems were separated from the alfalfa stems, and dry weight of the *C. campestris* stems were measured.

### 4.3. Enzymatic Assays 

ROS production and scavenging enzymes: activity of total Superoxide dismutase enzymes (EC 1.15.1.1) and its isoenzymes, catalase (EC 1.11.1.6), ascorbate peroxidase (EC 1.11.1.1), and glutathione peroxidase (EC 1.11.1.9) were measured based on the methods described by Sairam et al. [58], Aebi [59], Yoshimura et al. [60], and Velikova et al. [61] respectively. For enzyme extraction, leaf samples (0.5 g) were homogenized in ice-cold 0.1 M phosphate buffer (pH 7.5), containing 0.5 mM EDTA with pre-chilled pestle and mortar. Homogenates were transferred to centrifuge tubes and centrifuged at 4 °C for 15 min at 15,000× *g*.

SOD activity was estimated by recording the decrease in absorbance of superoxide-nitro blue tetrazolium complex by the enzyme. About 3 mL of reaction mixture, containing 0.1 mL of 200 mM methionine, 0.1 mL of 2.25 mM nitro-blue tetrazolium (NBT), 0.1 mL of 3 mM EDTA, 1.5 mL of 100 mM potassium phosphate buffer, 1 mL distilled water and 0.05 mL of enzyme extract, were mixed in test tubes in duplicate from each enzyme sample. Two tubes without enzyme extract were used as control. The reaction was started by adding 0.1 mL riboflavin (60 μM) and placing the tubes below a light source of two 15 W fluorescent lamps for 15 min. Reaction was stopped by switching off the light and covering the tubes with black cloth. A non-irradiated reaction mixture, which did not develop color was used as blank. Absorbance was recorded at 560 nm and one unit of enzyme activity was taken as the quantity of enzyme, which reduced the absorbance reading of samples to 50%, compared to the blank. 

CAT activity was measured in 3 mL reaction, containing 1.5 mL of 100 mM potassium phosphate buffer (pH = 7), 0.5 mL of 75 mM H_2_O_2_, and 0.05 mL enzyme solution. Reaction started by adding H_2_O_2_ and decrease in absorbance was recorded at 240 nm for 1 min. Enzyme activity was assessed by calculating the amount of H_2_O_2_ decomposed. 

APX activity was measured by monitoring the rate of ascorbate oxidation at 290 nm. The reaction mixture contained 25 mM phosphate buffer (pH = 7), 0.1 mM EDTA, 1 mM H_2_O_2_, 0.25 mM AsA, and the enzyme sample. No change in absorption was observed in the blank (reaction without AsA). 

GPX activity was assessed by recording the increased absorbance in the presence of oxidized glutathione (GSSG) and 5,5-dithiobis-2-nitrobenzoic acid (DTNB). The reaction mixture contained 1 mL of 0.2 M potassium phosphate buffer (pH = 7.5), 0.1 mM EDTA, 0.5 mL of 3 mM DTNB, 0.1 mL of 2 mM NADPH, 0.1 mL enzyme extract, and distilled water to make up a final volume of 2.9 mL. Reaction initiated by adding 0.1 mL of 2 mM GSSG. The increase in absorbance at 412 nm was recorded at 25 °C over a period of 5 min. 

The amount of hydrogen peroxide and the lipid peroxidation were quantified according to the method of Velikova et al. [61] and Stewart and Bewley [62], respectively.

### 4.4. Statistical Analysis

Before subjecting the collected data for statistical analyses and ANOVA, normality of data distribution were examined using Bartlett test. Data from each measurement were subjected for independent ANOVA analysis, followed by mean comparisons using Duncan Test at 5% probability level using MSTATC package [63]. Microsoft Excel and ggplot2 package in R environment [64] were used to draw graphs and boxplots.

## 5. Conclusions

The results of this study showed that the aqueous extracts of *N. meyeri* have allelopathic and phytotoxic effects the on germination and initial growth of *C. campestris* seedlings and the activity of antioxidant compounds. In comparison to control treatment, concentrations of H_2_O_2_ and MDA were increased in the *C. campestris* plants, which treated with catnip extract. This may be due to a reduction in measured antioxidant enzymes involved with oxidative stress. Except for the root extract application of other parts of catnip extracts, *C. campestris* reduced CAT, APX, GPX and SOD activity in early growth stages. Among the extracts of different organs, flower, leaf, and stem extracts exhibited stronger effects, compared to the root extract. Among the different stages of the *C. campestris* plant life, germination and early growth stages were more sensitive to the treatment with *N. meyeri* extracts. No apparent controlling effect was observed for extracts from various organs of *N. meyeri* after the alfalfa was infested by the *C. campestris*. Altogether, *N. meyeri* leaf and flower extracts can be considered as potent allelochemical agents to control *C. campestris* in the early growth stages.

## Figures and Tables

**Table 1:** Effect of extracts from different organs of *N. meyeri* on germination and vegetative traits in *C. campestris* seedling. Numbers represent mean and ±standard deviations. Different letters indicate statistically significant differences calculated by independent ANOVA analyses (*n* = 3, *p*-value < 0.05) for each phenotype followed by multiple mean comparison using Duncan method.

Flower	Leaf	Stem	Root	Control	Treatment
4.7 (±4.57) ^c^	10.3 (±6.47) ^c^	51.3 (±13.36) ^b^	71 (±14.78) ^a^	74 (±14.85) ^a^	Percentage of Germination
0.3 (±0.47) ^c^	0.4 (±0.77) ^c^	0.9 (±0.38) ^b^	1.1 (±10.84) ^ab^	1.2 (±1.56) ^a^	Seedling Root Length (cm)
2.6 (±2.43) ^d^	4.3 (±1.88) ^d^	9.7 (±3.16) ^c^	15.3 (±7.34) ^b^	19.3 (±8.43) ^a^	Seedling Shoot Length (cm)
1 (±0.14) ^d^	1 (±0.32) ^d^	6.7 (±3.79) ^c^	12.7 (±4.36) ^b^	16.7 (±5.37) ^a^	Seedling Dry Weight (mg)

**Table 2:** Effect of extracts from different organs of *N. meyeri* on *C. campestris* seedling establishment and parasitation of alfalfa plants. Different letters indicate statistically significant differences calculated by independent ANOVA analyses (n = 3, *p*-value < 0.05) for each phenotype followed by multiple mean comparison using Duncan method.

Flower	Leaf	Stem	Root	Control	Treatment
1 (±0.21) ^d^	2 (±0.17) ^d^	16.3 (±3.43) ^c^	19.3 (±3.11) ^b^	22 (±3.04) ^a^	Shoot Length (cm)
1 (±0.17) ^c^	1 (±0.15) ^c^	8.3 (±3.54) ^b^	10.7 (±3.07) ^b^	19 (±6.53) ^a^	Shoot Dry Weight (mg)
3.2 (±1.34) ^d^	14.7 (±3.11) ^c^	44.7 (±5.76) ^b^	62 (±10.46) ^a^	64.4 (±10.62) ^a^	Percentage of Emergence
2 (±0.034) ^d^	6 (±1.49) ^c^	15 (±2.27) ^c^	75 (±1.74) ^b^	100 (±0.001) ^a^	Percentage of alfalfa infestation

**Table 3:** Effect of *N. meyeri* extracts from different organs of on *C. campestris* and alfalfa vegetative traits and parasitation rate after attachment. Numbers represent means and ± standard deviations calculated for each phenotype, respectively. ANOVA analyses (n = 3, *p*-value < 0.05) were performed for each trait. Different letters indicate statistically significant differences. ns: not significant; Fw: fresh weight; Dw: dry weight.

	Control	Root	Stem	Leaf	Flower
Alfalfa Fw (gr)	29.67 (±5.21) ^ns^	28.52 (±6.41) ^ns^	25.38 (±7.36) ^ns^	27.67 (±4.42) ^ns^	26.25 (±8.08) ^ns^
Alfalfa Dw (gr)	2.25 (±0.26) ^ns^	2.46 (±0.21) ^ns^	2.14 (±0.37) ^ns^	2.23 (±0.25) ^ns^	1.98 (±0.33) ^ns^
Alfalfa height (cm)	32.77 (±4.15) ^ns^	29.31 (±5.41) ^ns^	27.75 (±3.20) ^ns^	28.13 (±6.73) ^ns^	26.82 (±3.58) ^ns^
Dodder Dw (gr)	0.97 (±0.16) ^a^	0.87 (±0.21) ^a^	0.93 (±0.15) ^a^	0.76 (±0.13) ^b^	0.84 (±0.21) ^a^
Parasitation rate	100%	100%	100%	100%	100%

## Data Availability

Not applicable.
